# Intake of meat and fish and risk of head–neck cancer subtypes in the Netherlands Cohort Study

**DOI:** 10.1007/s10552-017-0892-0

**Published:** 2017-04-05

**Authors:** Andy Perloy, Denise H. E. Maasland, Piet A. van den Brandt, Bernd Kremer, Leo J. Schouten

**Affiliations:** 1 Department of Epidemiology, GROW – School for Oncology and Developmental Biology, Maastricht University, Peter Debijeplein 1, 6229 HA Maastricht, The Netherlands; 2 Department of Otorhinolaryngology, Head and Neck Surgery, GROW – School for Oncology and Developmental Biology, Maastricht University Medical Center, Maastricht, The Netherlands

**Keywords:** Meat, Head–neck cancer, Prospective cohort studies, Risk factors, Fish

## Abstract

**Purpose:**

To date, the role of meat and fish intake in head–neck cancer (HNC) etiology is not well understood and prospective evidence is limited. This prompted us to study the association between meat, fish, and HNC subtypes, i.e., oral cavity cancer (OCC), oro- and hypopharyngeal cancer (OHPC), and laryngeal cancer (LC), within the Netherlands Cohort Study (NLCS).

**Methods:**

In 1986, 120,852 participants (aged 55–69 years) completed a baseline 150-item food frequency questionnaire (FFQ), from which daily meat and fish intake were calculated. After 20.3 years of follow-up, 430 HNC overall (134 OCC, 90 OHPC and 203 LC) cases and 4,111 subcohort members were found to be eligible for case–cohort analysis. Multivariate hazard ratios were calculated using Cox’s proportional hazards model within quartiles of energy-adjusted meat and fish intake.

**Results:**

Processed meat intake, but not red meat intake, was positively associated with HNC overall [HR(Q4 vs. Q1) = 1.46, 95% CI 1.06–2.00; *p*trend = 0.03]. Among HNC subtypes, processed meat was positively associated with OCC, while no associations were found with OHPC and LC. Fish intake was not associated with HNC risk. Tests for interaction did not reveal statistically significant interaction between meat, fish, and alcohol or smoking on HNC overall risk.

**Conclusions:**

In this large cohort study, processed meat intake was positively associated with HNC overall and HNC subtype OCC, but not with OHPC and LC.

**Electronic supplementary material:**

The online version of this article (doi:10.1007/s10552-017-0892-0) contains supplementary material, which is available to authorized users.

## Introduction

Head and neck cancer (HNC) includes among other cancers of the oral cavity (OCC), oro- and hypopharynx (OHPC), and larynx (LC), with roughly 90% being squamous cell carcinomas [1]. It is well established that tobacco and alcohol are strong risk factors for HNC and interact in a multiplicative way [2]. Recent incidence trends show a rise in human papillomavirus (HPV)-related oropharyngeal cancer cases, which is another important risk factor for HNC [3].

The role of diet in the etiology of HNC is less clear and most evidence is based on case–control studies [4]. High intake of vegetables and fruit is suggested to reduce HNC risk, which was recently confirmed in a prospective study [5]. In contrast, the evidence for meat, fish, and HNC is weak and inconsistent [1].

In various case–control studies [6–8], high intakes of red and processed meat have been positively associated with HNC, which may be explained by the carcinogenic compounds found in meat [9]. However, according to a recent meta-analysis of case–control studies, only high intake of processed, but not red meat may increase the risk of HNC subtypes oral cavity and oropharyngeal cancer [10].

So far, few prospective studies have investigated the relationship between meat and HNC, not showing a clear association [11–15]. Two studies [11, 12] differentiated between HNC subtypes, whereas others [13–15] only analyzed cancer of the upper digestive tract (HNC and esophageal cancer combined) and lacked sufficient cases to study HNC subtypes separately. Of the two studies that analyzed HNC subtypes, the NIH-AARP study found a positive association between red meat and laryngeal cancer [11]. In contrast, the European Prospective Investigation into Cancer and Nutrition (EPIC) study did not confirm this finding and found an increased risk of oral cavity and pharyngeal cancer with processed meat [12]. This discrepancy may be explained by differences in meat definition or levels of meat intake, and it also stresses the need for more prospective studies on this topic.

With regard to fish, case–control studies suggest that high intake of fish may reduce HNC risk [16, 17]. Fish contains long-chain omega-3 polyunsaturated fatty acids, which may protect against certain cancers (e.g., prostate and colon cancer) according to in vitro and in vivo studies [18]. However, previous cohort studies showed no association between fish and HNC [12, 13, 19].

To further clarify the association between meat, fish, and HNC subtypes, we conducted a study within the large Netherlands Cohort Study (NLCS) consisting of Dutch men and women between 55 and 69 years at baseline (1986). It is hypothesized that (*i*) HNC risk is higher in participants with high (total, red, and processed) meat intake compared to those with low intakes; (*ii*) HNC risk is lower in participants with high fish intake compared to those with low intakes; (*iii*) the risk is different for HNC subtypes OCC, OHPC, and LC; and (*iiii*) the major HNC risk factors alcohol and smoking interact with meat and fish consumption.

## Methods

### Study population and design

The Netherlands Cohort Study (NLCS) on diet and cancer is an ongoing prospective cohort study, initiated in 1986, with participants selected from 204 municipalities across the country. At baseline, 58,279 men and 62,573 women aged 55–69 years completed a self-administered questionnaire on dietary habits and other risk factors for cancer. Participants provided informed consent for study participation by completing and returning this questionnaire. Immediately after baseline, a subsample of 5,000 subjects was randomly selected from the full cohort for a case–cohort analysis approach. In the case–cohort design, complete data on exposures and confounders are only required for cases and subcohort members, which were chosen to efficiently process and analyze the detailed questionnaire (that also included open-ended questions). To accurately calculate the accumulated person-years of the subcohort, information on migration and vital status was firstly collected by contacting subcohort members and municipalities every 2 years and later on through linkage with the Dutch municipal population registries. Further details of the study have been described elsewhere [20]. The NLCS has been approved by the institutional review boards of the University Hospital Maastricht and TNO Nutrition and Food Research.

### Case identification and eligibility

During 20.3 years of follow-up, head–neck cancer (HNC) cases in the entire cohort were identified by annually repeated record linkage to the Netherlands Cancer Registry and PALGA, the nationwide histopathology and cytopathology data network [21]. Cases were classified as proposed by Hashibe et al. [22], according to the third version of the International Classification of Diseases for Oncology (ICD-O-3) [23]. Incident, microscopically confirmed squamous cell carcinoma cases of the head and neck were included. Participants with prevalent cancer other than skin cancer were excluded at baseline. Furthermore, participants were excluded if (1) dietary data were incomplete or inconsistent and/or (2) information was missing on confounders included in the multivariate model. Finally, 430 head–neck cancer cases and 4,111 subcohort members were available for analysis. Stratified by HNC subtypes, this included 134 oral cavity (ICD-O-3 C003-009, C020-C023, C030-C031, C039-C041, C048-C050, C060-C062, C068-C069), 90 oro- and hypopharyngeal (C019, C024, C051-C052, C090-C091, C098-C104, C108-C109, C129-C132, C138-C139), and 203 laryngeal (C320-329) cancer cases; 3 cases were oral cavity/pharynx unspecified or overlapping (C028-C029, C058-C059,C140-C142, C148).

### Dietary and covariate assessment

As part of the baseline questionnaire, a self-administered 150-item food frequency questionnaire (FFQ) assessed dietary habits over the last year prior to the study. The FFQ measured (monthly/weekly) frequency and serving sizes of meat and fish consumption, from which the mean daily intake (in grams) was calculated. Based on the sum of all corresponding meat items, meats were grouped into red meat (beef, pork, minced meat, and liver), processed meat (all types of meat preserved with nitrate salt, fermentation, or smoking), and chicken. To validate the FFQ, a 9-day dietary record method was completed by a random sample from the full cohort [24]. Spearman correlation coefficients were 0.46, 0.54, and 0.53 for, respectively, the food groups namely meat, processed meat, and fish. With respect to possible confounders, the baseline questionnaire gathered among other information on alcohol consumption, cigarette smoking (status, duration and frequency of smoking), vegetables and fruit consumption, total energy intake, body mass index (BMI, kilograms per square meter), non-occupational physical activity, education (four levels), and family history of HNC.

### Statistical analysis

Age- and sex-adjusted and multivariate-adjusted hazard ratios (HRs) were calculated using the Cox proportional hazards model with 95% confidence intervals (CIs). Standard errors were estimated using a robust variance estimator to adjust for additional variance introduced by sampling a subcohort from the full cohort [25]. Time-on-study was used as the underlying time-scale and defined as time from baseline to either diagnosis of HNC, death, emigration, loss to follow-up, or censoring (December 31, 2006), whichever came first.

Daily meat and fish intakes were adjusted for total energy intake (g/1,000 kcal) by the nutrient density method [26]. Subjects were divided into quartiles based on the sex-specific energy-adjusted intake distributions of the subcohort. For liver, chicken, and fish, there was a large group of non-consumers. Therefore, to be able to compare non-consumers with consumers, we intentionally assigned only non-consumers to the first category of these intake variables. Consequently, consumers were divided into one of the remaining three categories by using a tertile split. Meat and fish consumption was also modeled as a continuous variable by using increments of 2.5 g/1,000 kcal (liver), 5 g/1,000 kcal (processed meat), 10 g/1,000 kcal (fish, minced meat and chicken), 15 g/1,000 kcal (beef and pork), and 20 g/1,000 kcal (red meat). As previously described by Steffen et al. [12], these increments correspond approximately to the standard deviation from the mean value of each intake variable.

A priori confounders were age, sex, alcohol consumption (g/day), cigarette smoking status (never/former/current), frequency of cigarettes smoked per day, duration of smoking (years), and total energy intake (kcal/day). The continuous variables including cigarette smoking frequency and duration of smoking were centered in multivariate-adjusted models [27]. Potential confounders were assessed by backward elimination and included in the final model if the hazard ratio changed by more than 10%. BMI (kg/m^2^), non-occupational physical activity (min/day), level of education (four categories), family history of HNC (yes/no), and vegetable and fruit intake (g/1,000 kcal) were considered as potential confounders, but were not included in the final model according to this change-in-estimate criterion.

Tests for interaction were performed to examine effect modification by sex, alcohol, or cigarette smoking on HNC overall risk. For this purpose, alcohol intake was categorized into non-user, 0–15 g/day and ≥15 g/day and cigarette smoking was categorized as never, former, and current smoker. Hazard ratios for each stratum were calculated by adding cross-product terms between sex, alcohol or cigarette smoking and meat or fish in multivariate Cox models. The Wald test was used to test the significance of these interactions. We found no evidence for effect modification by sex (*p* for interaction > 0.05 for meat and fish) and therefore sexes were combined for analysis.

To test for a dose–response relationship across quartiles of meat and fish intake, tests for trend were performed by using the sex-specific energy-adjusted median of each quartile as a continuous variable. Heterogeneity between HNC subtypes was examined by using a bootstrap method developed for the case–cohort design [28]. Further details of this method have been described in detail elsewhere [2].

In sensitivity analyses, we tested for reverse causation by excluding cases in the first 2 years of follow-up. We also tested the effects of mutual adjustment for meat (e.g., processed meat adjusted for red meat and chicken). The proportional hazards (PH) assumption was assessed by using the scaled Schoenfeld residuals [29]. To further examine a possible violation, a smoothed residuals plot [30] and log-minus-log plot [31] were graphically assessed. With respect to HNC overall, the PH assumption was possibly violated for the current smoker covariate (that statistically significant interacted with time). Graphical assessment of the smoothed Schoenfeld residuals plot and log-minus-log plot did not show clear evidence of a violation. To verify this, a time-varying covariate (tvc) for current smoker was included in the model, which did not affect the hazard ratios. Hence, analysis was performed without tvc for current smoker. In case the PH assumption was violated for an exposure of interest, hazard ratios were calculated for different time periods (0–5, 5–10, and >10 years) of follow-up to see how the hazard ratio changed over time.

All statistical analyses were performed with STATA (version 13.1, StataCorp LP, College Station, TX, USA) and reported *p* values were two-sided, with *p* < 0.05 considered statistically significant.

## Results

### Baseline characteristics

In Table 1, baseline characteristics for cases and subcohort members are given, with dietary intake variables reported as nutrient density-adjusted variables (g/1,000 kcal). The mean age in the subcohort was 61.3 years. HNC cases, as compared to subcohort members, were predominantly men (especially LC cases; 93.1%). In the subcohort, the mean (SD) daily intake of red meat and processed meat was, respectively, 46.9 (22.9) and 6.5 (6.6) g/1,000 kcal. Female LC cases had lower daily red meat intake compared to that of female subcohort members and other female HNC subtype cases. Overall, the mean daily intake of red meat and processed meat did not differ meaningfully between subcohort members and cases. As compared to subcohort members, cases were more likely to be consumers of liver. Mean daily intake of fruit and vegetables was lower among cases than subcohort members, except for OCC cases. Furthermore, cases were more likely current smokers and more often consumers of alcohol. Moreover, cases who consumed alcohol had a higher mean daily intake of alcohol as compared to alcohol consumers from the subcohort.

**Table 1:** Baseline characteristics of eligible subcohort members and head–neck cancer cases in the Netherlands Cohort Study (1986–2006)

Baseline characteristics	Subcohort (*n* = 4,111)	Head–neck cancer cases			
		HNC overall (*n* = 430)	OCC (*n* = 134)	OHPC (*n* = 90)	LC (*n* = 203)
Age (years) mean (SD)	61.3 (4.2)	61.6 (4.1)	61.9 (4.2)	61.5 (4.0)	61.5 (4.0)
Sex: men (%)	49.2	77.7	57.5	74.4	93.1
Red meat (g/1,000 kcal) mean (SD)^a^	46.9 (22.9)	46.0 (21.5)	47.6 (22.3)	44.2 (22.9)	45.6 (20.4)
Men	44.6 (21.0)	45.1 (19.5)	46.1 (17.6)	41.6 (18.6)	46.0 (20.5)
Women	49.2 (24.3)	49.0 (27.2)	49.7 (27.4)	51.5 (31.7)	41.1 (19.7)
Processed meat (g/1,000 kcal) mean (SD)	6.5 (6.6)	7.3 (7.0)	7.2 (6.6)	6.6 (5.9)	7.8 (7.6)
Men	7.0 (6.8)	7.5 (6.7)	7.4 (6.7)	6.8 (5.8)	7.8 (7.0)
Women	6.1 (6.4)	6.8 (7.8)	6.9 (6.5)	6.0 (6.4)	7.7 (13.6)
Fish (g/1,000 kcal)^b^	7 (0–10)	7 (2–10)	8 (1–10)	9 (2–12)	7 (2–10)
Beef (g/1,000 kcal)^b^	14 (5–20)	14 (5–19)	16 (5–21)	12 (5–17)	13 (5–18)
Pork (g/1,000 kcal)^b^	20 (9–28)	20 (9–27)	19 (8–26)	20 (8–29)	20 (9–27)
Minced meat (g/1,000 kcal)^b^	10 (4–14)	10 (4–13)	10 (4–13)	10 (4–13)	10 (4–14)
Liver consumers (%)	35.9	43.0	43.0	45.6	41.9
Liver consumers: intake (g/1,000 kcal)^b^	3 (1–4)	3 (1–4)	3 (1–4)	3 (1–5)	3 (1–3)
Chicken (g/1,000 kcal)^b^	7 (2–10)	6 (2–9)	7 (2–11)	7 (2–10)	6 (2–9)
Fruit consumption (g/1,000 kcal)^b^	97 (49–130)	71 (24–99)	106 (52–142)	66 (16–85)	68 (25–97)
Vegetables consumption (g/1,000 kcal)^b^	107 (71–131)	97 (62–118)	108 (74–131)	95 (56–120)	90 (59–111)
Energy intake (kcal/day) mean (SD)	1,927 (513)	2,053 (550)	1,885 (589)	2,063 (470)	2,168 (526)
Men	2,169 (509)	2,175 (513)	2,102 (566)	2,184 (437)	2,205 (514)
Women	1,692 (395)	1,630 (462)	1,592 (487)	1,710 (384)	1,671 (440)
BMI (kg/m^2^) mean (SD)	25.0 (3.1)	24.8 (2.7)	24.9 (3.0)	24.4 (2.6)	25.0 (2.6)
Level of education (%)					
Primary school	28.2	27.9	21.6	22.5	33.8
Lower vocational	22.0	17.8	16.4	19.1	18.4
High school	35.6	35.1	39.6	37.1	31.8
Higher vocational/university	14.2	19.2	22.4	21.4	15.9
Physical activity, non-occupational (min/day)^b^	73 (34–94)	71 (34–90)	67 (34–86)	67 (30–88)	75 (39–94)
Cigarette smoking status (%)					
Never smoker	36.9	13.5	29.1	7.8	5.9
Former smoker	36.0	29.5	25.4	28.9	33.0
Current smoker	27.1	57.0	45.5	63.3	61.1
Ever cigarette smokers					
Frequency of cigarette smoking (*n*/day)	15.3 (10.3)	19.5 (10.8)	20.4 (11.8)	21.1 (12.6)	18.4 (9.4)
Duration of cigarette smoking (years)	31.6 (12.3)	38.7 (9.8)	36.9 (10.0)	38.1 (10.2)	39.9 (9.6)
Pack-years of cigarette smoking (*n*)	22.6 (17.7)	34.2 (21.0)	34.9 (23.4)	36.3 (23.3)	33.0 (18.7)
Alcohol consumers (%)	76.3	89.5	90.3	86.7	90.2
Alcohol consumers: ethanol intake (g/day) mean (SD)	13.5 (15.0)	26.8 (26.1)	27.0 (27.4)	35.2 (31.3)	23.1 (21.9)
Family history of HNC (%)	1.9	1.9	1.5	1.1	2.0

*HNC* head–neck cancer, *OCC* oral cavity cancer, *OHPC* oro- and hypopharyngeal cancer, *LC* laryngeal cancer, *SD* standard deviation

^a^Weight of red meat is based on raw meat weights

^b^The median and interquartile range are shown, because of a right-skewed distribution

### Main analyses

Results of the age- and sex-adjusted analysis (Supplemental Table S1) were generally comparable to that of the multivariate analysis, but hazard ratios were generally lower in the multivariate model.

No associations were found between a high intake of red meat and HNC risk in multivariate analysis (Table 2). High intake of processed meat was positively associated with HNC overall and HNC subtype OCC. Comparing the highest with the lowest quartile of processed meat intake, results were statistically significant in both HNC overall (HR 1.46, 95% CI 1.06–2.00; *p*trend = 0.03) and OCC (HR 1.88, 95% CI 1.11–3.18; *p*trend = 0.04). After processed meat was analyzed as a continuous variable (5 g/1,000 kcal increment), a statistically significant positive association was found only in OCC (HR 1.13, 95% CI 1.00–1.27). No association was found between processed meat and OHPC. For LC, increased hazard ratios were observed with processed meat intake, but they did not reach statistical significance and no statistically significant trend across quartiles of processed meat intake was observed. High intake of fish was not associated with HNC risk.

**Table 2:** Multivariate-adjusted hazard ratios (HRs) and 95% confidence intervals (CIs) of head–neck cancer subtypes by intake of red meat, processed meat, fish and types of fresh meat in the Netherlands Cohort Study (1986–2006)

Dietary exposure	Subcohort (*n* = 4,111)			Head–neck cancer cases							
				HNC overall (*n* = 430)		OCC (*n* = 134)		OHPC (*n* = 90)		LC (*n* = 203)	
	Median intake (g/1,000 kcal)^a^			*n* cases	HR (95% CI)^b^	*n* cases	HR (95% CI)^b^	*n* cases	HR (95% CI)^b^	*n* cases	HR (95% CI)^b^
	Male	Female	Person-years								
Red meat											
Q1	24	24	17,669	104	1.00 (ref)	31	1.00 (ref)	28	1.00 (ref)	45	1.00 (ref)
Q2	36	40	17,391	106	1.04 (0.76–1.41)	34	1.10 (0.66–1.83)	16	0.58 (0.31–1.09)	56	1.27 (0.84–1.94)
Q3	48	54	17,201	105	0.88 (0.64–1.22)	34	0.90 (0.53–1.52)	22	0.62 (0.32–1.21)	47	1.00 (0.64–1.55)
Q4	68	76	17,170	115	0.92 (0.67–1.27)	35	0.81 (0.48–1.37)	24	0.68 (0.36–1.30)^e^	55	1.16 (0.75–1.79)
*p* trend					0.47		0.32		0.35		0.80
Continuous, per 20 g/1,000 kcal			69,431		0.94 (0.84–1.04)^e^		0.93 (0.79–1.09)		0.83 (0.65–1.05)^f,g^		0.99 (0.87–1.14)
Processed meat											
Q1	1	0	17,360	92	1.00 (ref)	27	1.00 (ref)	22	1.00 (ref)	43	1.00 (ref)
Q2	4	3	17,380	118	1.20 (0.87–1.67)	38	1.46 (0.85–2.51)	24	1.01 (0.54–1.88)^e^	55	1.18 (0.77–1.82)
Q3	7	6	17,440	87	0.92 (0.65–1.31)	26	1.02 (0.56–1.84)^e^	20	0.86 (0.43–1.71)	40	0.89 (0.56–1.41)^e^
Q4	14	13	17,250	133	1.46 (1.06–2.00)	43	1.88 (1.11–3.18)	24	1.12 (0.57–2.20)^e^	65	1.39 (0.92–2.11)
*p* trend					0.03		0.04		0.75		0.16
Continuous, per 5 g/1,000 kcal			69,431		1.07 (0.99–1.16)		1.13 (1.00–1.27)		1.00 (0.83–1.19)		1.07 (0.96–1.19)
Fish											
C1^c^	0	0	19.830	94	1.00 (ref)	33	1.00 (ref)	19	1.00 (ref)	41	1.00 (ref)
C2	3	3	16,729	101	1.09 (0.79–1.51)	30	1.02 (0.61–1.72)	16	0.77 (0.37–1.58)	54	1.30 (0.83–2.02)
C3	7	8	16,706	122	1.25 (0.91–1.70)^e^	40	1.26 (0.78–2.05)^e^	28	1.28 (0.68–2.42)	54	1.25 (0.81–1.93)
C4	14	16	16,166	113	1.09 (0.79–1.49)^e^	31	0.86 (0.51–1.43)	27	1.16 (0.62–2.15)	54	1.25 (0.80–1.94)^e^
*p* trend					0.68		0.53		0.39		0.50
Continuous, per 10 g/1,000 kcal			69,431		1.00 (0.88–1.12)		0.98 (0.79–1.21)		1.12 (0.91–1.36)		0.96 (0.82–1.12)
Beef											
Q1	2	2	17,530	104	1.00 (ref)	31	1.00 (ref)	22	1.00 (ref)	51	1.00 (ref)
Q2	7	8	17,467	123	1.09 (0.81–1.48)	34	1.02 (0.62–1.70)	34	1.37 (0.77–2.44)	54	1.00 (0.66–1.52)
Q3	14	15	17,335	93	0.73 (0.53–1.01)	25	0.62 (0.36–1.08)	15	0.50 (0.24–1.03)	51	0.91 (0.60–1.38)
Q4	26	29	17,099	110	0.93 (0.68–1.27)	44	1.09 (0.66–1.80)	19	0.66 (0.33–1.33)	47	0.92 (0.60–1.41)
*P* trend					0.35		0.85		0.06		0.67
Continuous, per 15 g/1,000 kcal			69,431		0.96 (0.82–1.11)^e^		1.08 (0.88–1.33)		0.70 (0.48–1.04)		0.96 (0.78–1.19)
Pork											
Q1	4	3	17,610	108	1.00 (ref)	36	1.00 (ref)	24	1.00 (ref)	47	1.00 (ref)
Q2	13	14	17,250	97	0.78 (0.56–1.08)	28	0.67 (0.38–1.17)	22	0.71 (0.38–1.34)	47	0.92 (0.59–1.42)
Q3	21	23	17,278	119	1.00 (0.74–1.34)	44	1.17 (0.73–1.86)	17	0.62 (0.33–1.18)	58	1.10 (0.73–1.66)
Q4	35	40	17,292	106	0.76 (0.55–1.05)	26	0.52 (0.29–0.92)	27	0.79 (0.43–1.45)	51	0.92 (0.59–1.42)
*P* trend					0.20		0.07		0.58		0.80
Continuous, per 15 g/1,000 kcal			69,431		0.92 (0.82–1.03)		0.84 (0.71–1.00)		0.90 (0.72–1.14)		0.98 (0.85–1.14)
Minced meat											
Q1	2	1	17,385	100	1.00 (ref)	30	1.00 (ref)	20	1.00 (ref)	50	1.00 (ref)
Q2	6	6	17,298	113	1.02 (0.75–1.40)	29	0.88 (0.51–1.51)	28	1.17 (0.64–2.16)	54	1.00 (0.66–1.53)
Q3	10	11	17,602	116	1.12 (0.82–1.53)	46	1.46 (0.89–2.39)	22	1.01 (0.53–1.95)	48	0.96 (0.62–1.47)
Q4	19	20	17,146	101	0.99 (0.72–1.35)	29	0.93 (0.55–1.58)	20	0.98 (0.51–1.87)	51	1.01 (0.66–1.53)
*P *trend					0.98		0.86		0.86		0.99
Continuous, per 10 g/1,000 kcal			69,431		1.03 (0.93–1.15)		0.98 (0.83–1.15)		0.99 (0.79–1.24)		1.08 (0.93–1.26)
Liver											
C1^c^	0	0	44,704	245	1.00 (ref)	77	1.00 (ref)	49	1.00 (ref)	118	1.00 (ref)
C2^d^	2	2	24,726	185	1.22 (0.97–1.53)	57	1.32 (0.91–1.92)	41	1.34 (0.85–2.10)	85	1.12 (0.83–1.52)
Continuous, per 2.5 g/1,000 kcal			69,431		1.08 (0.98–1.19)		1.12 (0.98–1.27)		1.14 (0.97–1.33)		1.01 (0.87–1.18)
Chicken											
C1^c^	0	0	16,112	96	1.00 (ref)	31	1.00 (ref)	20	1.00 (ref)	45	1.00 (ref)
C2	3	3	17,970	138	1.38 (1.02–1.87)	43	1.43 (0.88–2.33)	26	1.27 (0.70–2.34)	67	1.38 (0.91–2.10)
C3	7	8	17,625	101	0.85 (0.60–1.20)	30	0.78 (0.44–1.40)	24	0.84 (0.41–1.71)	46	0.88 (0.56–1.40)
C4	12	16	17,722	95	0.88 (0.64–1.22)	30	0.83 (0.49–1.40)	20	0.89 (0.46–1.72)	45	0.95 (0.61–1.48)^e^
*p* trend					0.08		0.19		0.58		0.28
Continuous, per 10 g/1,000 kcal			69,431		0.91 (0.79–1.06)		0.93 (0.73–1.18)		0.93 (0.70–1.22)		0.90 (0.73–1.11)

*HNC* head–neck cancer, OCC oral cavity cancer, *OHPC* oro- and hypopharyngeal cancer, *LC* laryngeal cancer, *Q* quartile, *C* category

^a^Nutrient density-adjusted intakes

^b^Adjusted for age (years), sex, cigarette smoking status (never/former/current), frequency of smoking (number of cigarettes per day; centered), duration of smoking (number of years; centered), alcohol consumption (grams ethanol per day), and total energy intake (kcal/day)

^c^Non-consumers

^d^Intake > 0 g/day

^e^Possible violation of the proportional hazards assumption; no statistically significant interaction with time (*p* value ≥ 0.05)

^f^Possible violation of the proportional hazards assumption; statistically significant interaction with time (*p* value < 0.05)

^g^Hazard ratios were calculated for different time periods of follow-up: 0–5 years: HR 0.81 (95% CI 0.56–1.18); 5–10 years: HR 0.51 (95% CI 0.32–0.82) and >10 years: HR 1.03 (95% CI 0.73–1.45)

Multivariate analysis of individual meats (Table 2) showed no association between high intake of beef and HNC risk. When comparing the highest with the lowest quartile of pork intake, a statistically significant inverse association was found between pork and OCC (HR 0.52, 95% CI 0.29–0.92). No statistically significant inverse trend across quartiles of pork intake was observed. When tested as a continuous variable (15 g/1,000 kcal increment), pork remained statistically significantly inversely associated with OCC (HR 0.84, 95% CI 0.71–1.00). Pork was not associated with HNC overall, OHPC, and LC. High intake of minced meat was not associated with HNC risk. Liver was consistently positive, but not statistically significantly associated with HNC risk. With respect to chicken, point estimates (Q4 vs. Q1 and continuous) were consistently below unity, but never reached statistically significant levels.

Tests for heterogeneity showed no statistically significant difference between HNC subtypes except for beef continuously analyzed (data not shown).

### Interaction with alcohol and smoking

Within the subgroup former smokers, those in the highest quartile of red meat intake had a statistically significant decreased risk of HNC overall (Supplemental Figure S1; HR 0.54, 95% CI 0.32–0.91). The test for multiplicative interaction between cigarette smoking status and quartiles of red meat intake revealed no statistically significant interaction (*p* for interaction = 0.13). Among current smokers, those in the 3rd quartile of fish intake had a statistically significant increased risk of HNC overall (Supplemental Figure S2; HR 1.69, 95% CI 1.07–2.66). No statistically significant multiplicative interaction was observed between cigarette smoking status and quartiles of fish intake (*p* for interaction = 0.17). Participants who drank ≥ 15 grams of alcohol per day and were in the highest quartile of processed meat intake had a statistically significant increased risk of HNC overall (Supplemental Figure S3; HR 1.76, 95% CI 1.07–2.91). The test for multiplicative interaction between alcohol consumption and quartiles of processed meat intake did not show statistically significant interaction (*p* for interaction = 0.39).

No statistically significant multiplicative interactions were found between quartiles of either red meat or fish and alcohol consumption (data not shown; Supplemental Figures S4 to S5). Also, intake of processed meat did not statistically significantly interact with cigarette smoking status (data not shown; Supplemental Figure S6).

### Sensitivity analyses

In sensitivity analyses, we tested for reverse causation by excluding cases in the first two years of follow-up. However, this did not impact the results (data not shown). We also tested the effects of mutual adjustment for meat intake, but results remained unchanged (data not shown).

## Discussion

In this large prospective study of 120,852 men and women, high intake of processed meat, but not red meat, was positively associated with HNC overall. Among HNC subtypes, processed meat was positively associated with OCC, while no associations were found with OHPC and LC. High intake of fish was not associated with HNC risk. Individual meats showed no clear associations with HNC risk. Finally, alcohol and smoking did not modify the association between meat or fish and HNC overall risk.

Various case–control studies have investigated the red meat–HNC relationship, finding inconsistent results [4, 10]. Moreover, studies did not always clearly define red meat. In our study, red meat included all types of red meat except processed red meat. Few other prospective studies examined the association between red meat and HNC [11, 12]. In the NIH-AARP study [11], red meat included processed red meat, which appeared to increase the risk of LC [HR(Q5 vs. Q1) = 1.43, 95% CI 0.99–2.07]. However, after they excluded processed red meat in sensitivity analysis, it was not mentioned whether this result persisted. Similar to our results, the EPIC study [12] did not find an association with red meat. Thus, based on results from prospective observational studies, there seems to be no clear evidence for a positive association between red meat and HNC.

We observed a positive association between processed meat and HNC overall [HR(Q4 vs. Q1) = 1.46, 95% CI 1.06–2.00]. In the EPIC study [12], a positive association was found between processed meat and cancer of the upper aerodigestive tract (UADT). With regard to HNC subtypes, processed meat was positively associated with OCC in our study. In a meta-analysis of case–control studies [10], processed meat was positively associated with OCC and oropharyngeal cancer combined, but these HNC subtypes were not separately analyzed as was done in our study. The two other prospective studies [11, 12] also combined OCC with (oro-)pharyngeal cancer. In the NIH-AARP study [11], no association was found between processed meat and OCC and pharyngeal cancer combined, while the EPIC study [12] showed a positive association for this relationship. For HNC subtypes OHPC and LC, we did not find any association with processed meat. With regard to LC, the two other prospective studies also found no clear association with processed meat [11, 12]. Despite the fact the processed meat was differentially associated with HNC subtypes in our study, we did not observe statistically significant heterogeneity among HNC subtypes (except for beef; continuously analyzed). This may be due to a lack of power in our analysis.

No associations between fish and HNC were found in this study, which is in line with other prospective cohort studies [12, 13, 19]. However, these studies and our study did not differentiate between the types of fish (lean/fatty fish) consumed. Consequently, this may have influenced results and limited interpretation with regard to fish and HNC [32].

With respect to individual meats, we found no associations between beef and HNC. In contrast, one other cohort study found a positive association between beef and cancer of the UADT [13]. That study, however, included 71 cases and intake was only measured by frequency of consumption. For pork, we found an unexpected inverse association with OCC, which may reflect a chance finding. In case–control studies, pork was positively associated with HNC overall [4]. The consumption of liver was consistently positive but not statistically significantly associated with HNC in our study. Other studies found inconsistent results for liver [33, 34]. We did not find an association between chicken and HNC, which is in line with results of the EPIC study [12]. In the NIH-AARP study, intake of chicken was inversely associated with LC, but in women only [19]. Overall, there appears to be no clear evidence for associations between individual meats and HNC.

We found no evidence for an interaction between meat, fish and alcohol or smoking on HNC overall risk. However, we observed an increased HNC overall risk for participants who drank ≥ 15 g of alcohol per day and were in the upper quartile of processed meat intake. Given the low number of cases across strata (especially in the non-users stratum), we possibly had limited power to detect a significant deviation from the multiplicative model. Also, an inverse association with HNC overall was shown for former smokers that were in the highest quartile of red meat intake, which we cannot explain and may possibly reflect a chance finding. In contrast to our results, the EPIC study found high intake of processed meat to be positively associated with cancer of the UADT among smokers [12]. No other prospective cohort studies have examined the interaction between meat, fish and alcohol or smoking on HNC overall risk.

Two mechanisms have been described that relate high intake of processed meat to the development of various cancers. First, processed meat contains nitrate and nitrite, which leads to the (exogenous) formation of *N*-nitroso compounds that may have carcinogenic effects [35]. Second, processed meat contains several carcinogenic substances (such as heterocyclic amines and polycyclic aromatic hydrocarbons), which can be formed during high-temperature cooking [36]. However, the latter mechanism also applies to red meat, for which we found no association with HNC. Furthermore, the (endogenous) formation of *N*-nitroso compounds is also possible for red meat, which is influenced by its haem content [9]. Therefore, the previously discussed mechanisms do not clearly explain our findings regarding processed meat intake. While there is still limited prospective evidence for a positive association between processed meat and HNC, it is probably too premature to speculate about other possible mechanisms that may explain our findings. However, our result regarding processed meat at least suggests that processed meat as compared to red meat contains specific carcinogenic substances that may contribute to an increased HNC risk.

The strengths of our study are the prospective nature, the detailed baseline assessment of exposures and covariates, the (>96%) completeness [37] and duration of cancer follow-up, and a sufficient number of HNC (subtype) cases available for analysis. Of course, we also have to take our study limitations into account. First, participants completed a one-time baseline questionnaire and could have changed their dietary pattern during follow-up, possibly resulting in misclassification of meat intake. However, the FFQ in our study has been shown to reflect dietary intake over (at least) a 5-year period [38]. Second, we cannot rule out residual confounding from measured covariates, although important HNC confounders such as alcohol consumption and smoking were intensively addressed by our questionnaire. In addition, we have to consider the possibility of residual confounding from unmeasured confounders, such as HPV infection [2]. HPV, however, is especially associated with oropharyngeal cancer [39] and it is doubtful whether meat intake would have correlated with HPV exposure in this study.

In summary, in this large Dutch cohort study, processed meat was positively associated with HNC overall and HNC subtype OCC, but not with OHPC and LC. Future prospective studies on meat intake and HNC (subtypes) are needed to elaborate on our findings.

## Electronic supplementary material

Below is the link to the electronic supplementary material.

Supplementary material 1 (DOCX 673 KB)
