# Supplementary material for: Intake of meat and fish and risk of head–neck cancer subtypes in the Netherlands Cohort Study
Source: Cancer Causes Control. 2017 Apr 5;28(6):647–56. doi: 10.1007/s10552-017-0892-0 (PMC5400785; doi:10.1007/s10552-017-0892-0)
Supplement: Supplementary file 1 — Supplementary material 1 (DOCX 673 KB) [file 10552_2017_892_MOESM1_ESM.docx]

| **Supplemental Table S1.** Age- and sex-adjusted hazard ratios (HRs) and 95 % confidence intervals (CIs) of head-neck cancer subtypes by intake of red meat, processed meat, fish and types of fresh meat in the Netherlands Cohort Study (1986-2006) | | | | | | | | | | | | | | | |
| --- | --- | --- | --- | --- | --- | --- | --- | --- | --- | --- | --- | --- | --- | --- | --- |
|  |  | | |  | Head-neck cancer cases | | | | | | | | | | |
|  | Subcohort (*n* = 4,111) | | |  | HNC overall (*n* = 430) | |  | OCC (*n* = 134) | |  | OHPC (*n* = 90) | |  | LC (*n* = 203) | |
|  | Median intake  (g/1,000 kcal)^a^ | |  |  |  | | |  | | |  | |  | | |
| Dietary exposure | Male | Female | Person-years |  | *n* cases | HR (95 % CI)^b^ |  | *n* cases | HR (95 % CI)^b^ |  | *n* cases | HR (95 % CI)^b^ |  | *n* cases | HR (95 % CI)^b^ |
| Red meat |  |  |  |  |  |  |  |  |  |  |  |  |  |  |  |
| Q1 | 24 | 24 | 17,669 |  | 104 | 1.00 (ref) |  | 31 | 1.00 (ref) |  | 28 | 1.00 (ref) |  | 45 | 1.00 (ref) |
| Q2 | 36 | 40 | 17,391 |  | 106 | 1.04 (0.78-1.39) |  | 34 | 1.12 (0.68-1.83) |  | 16 | 0.58 (0.31-1.09) |  | 56 | 1.27 (0.84-1.92) |
| Q3 | 48 | 54 | 17,201 |  | 105 | 1.05 (0.79-1.41) |  | 34 | 1.14 (0.69-1.86) |  | 22 | 0.82 (0.46-1.44) |  | 47 | 1.09 (0.71-1.67) |
| Q4 | 68 | 76 | 17,170 |  | 115 | 1.15 (0.86-1.53) |  | 35 | 1.17 (0.72-1.92) |  | 24 | 0.89 (0.51-1.55) |  | 55 | 1.27 (0.84-1.92) |
| *p*trend |  |  |  |  |  | 0.35 |  |  | 0.55 |  |  | 0.96 |  |  | 0.41 |
| Continuous, per 20 g/1,000 kcal | |  | 69,431 |  |  | 1.03 (0.94-1.13) |  |  | 1.06 (0.91-1.22) |  |  | 0.95 (0.77-1.16)^f^ |  |  | 1.05 (0.92-1.19) |
| Processed meat |  |  |  |  |  |  |  |  |  |  |  |  |  |  |  |
| Q1 | 1 | 0 | 17,360 |  | 92 | 1.00 (ref) |  | 27 | 1.00 (ref) |  | 22 | 1.00 (ref) |  | 43 | 1.00 (ref) |
| Q2 | 4 | 3 | 17,380 |  | 118 | 1.33 (0.99-1.78) |  | 38 | 1.48 (0.89-2.43) |  | 24 | 1.12 (0.62-2.01)^e^ |  | 55 | 1.32 (0.87-2.00) |
| Q3 | 7 | 6 | 17,440 |  | 87 | 0.96 (0.70-1.31) |  | 26 | 0.99 (0.58-1.71) |  | 20 | 0.92 (0.50-1.70) |  | 40 | 0.94 (0.60-1.47)^e^ |
| Q4 | 14 | 13 | 17,250 |  | 133 | 1.51 (1.13-2.01) |  | 43 | 1.70 (1.04-2.78) |  | 24 | 1.13 (0.62-2.04) |  | 65 | 1.56 (1.05-2.33) |
| *p*trend |  |  |  |  |  | 0.02 |  |  | 0.08 |  |  | 0.81 |  |  | 0.06 |
| Continuous, per 5 g/1,000 kcal | |  | 69,431 |  |  | 1.07 (1.00-1.15) |  |  | 1.09 (0.97-1.21) |  |  | 0.99 (0.85-1.15) |  |  | 1.10 (0.99-1.21) |
| Fish |  |  |  |  |  |  |  |  |  |  |  |  |  |  |  |
| C1^c^ | 0 | 0 | 19.830 |  | 94 | 1.00 (ref) |  | 33 | 1.00 (ref) |  | 19 | 1.00 (ref) |  | 41 | 1.00 (ref) |
| C2 | 3 | 3 | 16,729 |  | 101 | 1.18 (0.88-1.59) |  | 30 | 1.07 (0.65-1.77) |  | 16 | 0.93 (0.48-1.83) |  | 54 | 1.37 (0.90-2.09) |
| C3 | 7 | 8 | 16,706 |  | 122 | 1.41 (1.06-1.88)^e^ |  | 40 | 1.41 (0.89-2.26)^e^ |  | 28 | 1.62 (0.89-2.93) |  | 54 | 1.36 (0.89-2.07) |
| C4 | 14 | 16 | 16,166 |  | 113 | 1.36 (1.01-1.82)^e^ |  | 31 | 1.13 (0.69-1.86) |  | 27 | 1.62 (0.90-2.95) |  | 54 | 1.42 (0.93-2.16)^e^ |
| *P*trend |  |  |  |  |  | 0.04 |  |  | 0.56 |  |  | 0.04 |  |  | 0.18 |
| Continuous, per 10 g/1,000 kcal | |  | 69,431 |  |  | 1.11 (1.00-1.23) |  |  | 1.12 (0.93-1.35) |  |  | 1.27 (1.07-1.50) |  |  | 1.03 (0.90-1.19) |
| Beef |  |  |  |  |  |  |  |  |  |  |  |  |  |  |  |
| Q1 | 2 | 2 | 17,530 |  | 104 | 1.00 (ref) |  | 31 | 1.00 (ref) |  | 22 | 1.00 (ref) |  | 51 | 1.00 (ref) |
| Q2 | 7 | 8 | 17,467 |  | 123 | 1.18 (0.89-1.56) |  | 34 | 1.08 (0.66-1.78)^e^ |  | 34 | 1.54 (0.89-2.65) |  | 54 | 1.06 (0.71-1.58) |
| Q3 | 14 | 15 | 17,335 |  | 93 | 0.89 (0.66-1.20) |  | 25 | 0.80 (0.46-1.36) |  | 15 | 0.68 (0.35-1.32) |  | 51 | 1.00 (0.67-1.51) |
| Q4 | 26 | 29 | 17,099 |  | 110 | 1.04 (0.78-1.39) |  | 44 | 1.38 (0.86-2.23) |  | 19 | 0.86 (0.46-1.60) |  | 47 | 0.91 (0.61-1.38) |
| *P*trend |  |  |  |  |  | 0.81 |  |  | 0.24 |  |  | 0.18 |  |  | 0.60 |
| Continuous, per 15 g/1,000 kcal | |  | 69,431 |  |  | 1.01 (0.88-1.15)^e^ |  |  | 1.18 (0.98-1.41) |  |  | 0.80 (0.58-1.10) |  |  | 0.96 (0.79-1.17) |
| Pork |  |  |  |  |  |  |  |  |  |  |  |  |  |  |  |
| Q1 | 4 | 3 | 17,610 |  | 108 | 1.00 (ref) |  | 36 | 1.00 (ref) |  | 24 | 1.00 (ref) |  | 47 | 1.00 (ref) |
| Q2 | 13 | 14 | 17,250 |  | 97 | 0.95 (0.71-1.27) |  | 28 | 0.82 (0.49-1.35) |  | 22 | 0.96 (0.54-1.72) |  | 47 | 1.06 (0.69-1.61) |
| Q3 | 21 | 23 | 17,278 |  | 119 | 1.16 (0.88-1.54) |  | 44 | 1.28 (0.82-2.02) |  | 17 | 0.74 (0.40-1.40) |  | 58 | 1.31 (0.88-1.96) |
| Q4 | 35 | 40 | 17,292 |  | 106 | 1.05 (0.78-1.40) |  | 26 | 0.77 (0.46-1.29) |  | 27 | 1.19 (0.68-2.09) |  | 51 | 1.16 (0.76-1.76) |
| *P*trend |  |  |  |  |  | 0.59 |  |  | 0.57 |  |  | 0.61 |  |  | 0.41 |
| Continuous, per 15 g/1,000 kcal | |  | 69,431 |  |  | 1.01 (0.92-1.10) |  |  | 0.94 (0.81-1.09) |  |  | 1.01 (0.84-1.21) |  |  | 1.05 (0.92-1.19) |
| Minced meat |  |  |  |  |  |  |  |  |  |  |  |  |  |  |  |
| Q1 | 2 | 1 | 17,385 |  | 100 | 1.00 (ref) |  | 30 | 1.00 (ref) |  | 20 | 1.00 (ref) |  | 50 | 1.00 (ref) |
| Q2 | 6 | 6 | 17,298 |  | 113 | 1.14 (0.85-1.52) |  | 29 | 0.97 (0.58-1.64) |  | 28 | 1.41 (0.79-2.52) |  | 54 | 1.08 (0.72-1.62) |
| Q3 | 10 | 11 | 17,602 |  | 116 | 1.17 (0.87-1.56) |  | 46 | 1.55 (0.97-2.49) |  | 22 | 1.10 (0.60-2.04) |  | 48 | 0.96 (0.64-1.46) |
| Q4 | 19 | 20 | 17,146 |  | 101 | 1.04 (0.77-1.40) |  | 29 | 1.00 (0.60-1.69) |  | 20 | 1.03 (0.55-1.93) |  | 51 | 1.04 (0.69-1.57) |
| *P*trend |  |  |  |  |  | 0.90 |  |  | 0.72 |  |  | 0.79 |  |  | 0.96 |
| Continuous, per 10 g/1,000 kcal | |  | 69,431 |  |  | 1.03 (0.93-1.14) |  |  | 0.99 (0.84-1.17) |  |  | 0.98 (0.79-1.22) |  |  | 1.07 (0.93-1.24) |

| **Supplemental Table S1.** Age- and sex-adjusted hazard ratios (HRs) and 95 % confidence intervals (CIs) of head-neck cancer subtypes by intake of red meat, processed meat, fish and types of fresh meat in the Netherlands Cohort Study (1986-2006) | | | | | | | | | | | | | | | |
| --- | --- | --- | --- | --- | --- | --- | --- | --- | --- | --- | --- | --- | --- | --- | --- |
|  |  | | |  | Head-neck cancer cases | | | | | | | | | | |
|  | Subcohort (*n* = 4,111) | | |  | HNC overall (*n* = 430) | |  | OCC (*n* = 134) | |  | OHPC (*n* = 90) | |  | LC (*n* = 203) | |
|  | Median intake  (g/1,000 kcal)^a^ | |  |  |  | | |  | | |  | |  | | |
| Dietary exposure | Male | Female | Person-years |  | *n* cases | HR (95 % CI)^b^ |  | *n* cases | HR (95 % CI)^b^ |  | *n* cases | HR (95 % CI)^b^ |  | *n* cases | HR (95 % CI)^b^ |
| Liver |  |  |  |  |  |  |  |  |  |  |  |  |  |  |  |
| C1^c^ | 0 | 0 | 44,704 |  | 245 | 1.00 (ref) |  | 77 | 1.00 (ref) |  | 49 | 1.00 (ref) |  | 118 | 1.00 (ref) |
| C2^d^ | 2 | 2 | 24,726 |  | 185 | 1.31 (1.07-1.61) |  | 57 | 1.38 (0.97-1.97) |  | 41 | 1.46 (0.96-2.23) |  | 85 | 1.19 (0.89-1.60) |
| Continuous, per 2.5 g/1,000 kcal | |  | 69,431 |  |  | 1.11 (1.02-1.21) |  |  | 1.14 (1.01-1.29) |  |  | 1.16 (1.00-1.35) |  |  | 1.03 (0.90-1.19) |
| Chicken |  |  |  |  |  |  |  |  |  |  |  |  |  |  |  |
| C1^c^ | 0 | 0 | 16,112 |  | 96 | 1.00 (ref) |  | 31 | 1.00 (ref) |  | 20 | 1.00 (ref) |  | 45 | 1.00 (ref) |
| C2 | 3 | 3 | 17,970 |  | 138 | 1.30 (0.98-1.73) |  | 43 | 1.26 (0.79-2.01) |  | 26 | 1.18 (0.65-2.13) |  | 67 | 1.35 (0.91-2.01) |
| C3 | 7 | 8 | 17,625 |  | 101 | 1.00 (0.74-1.35) |  | 30 | 0.93 (0.56-1.54) |  | 24 | 1.13 (0.62-2.07) |  | 46 | 0.97 (0.63-1.48) |
| C4 | 12 | 16 | 17,722 |  | 95 | 0.92 (0.68-1.24) |  | 30 | 0.90 (0.54-1.49) |  | 20 | 0.93 (0.49-1.73) |  | 45 | 0.93 (0.61-1.43) |
| *p*trend |  |  |  |  |  | 0.22 |  |  | 0.45 |  |  | 0.89 |  |  | 0.28 |
| Continuous, per 10 g/1,000 kcal | |  | 69,431 |  |  | 0.94 (0.82-1.08) |  |  | 0.99 (0.79-1.24) |  |  | 0.97 (0.76-1.24) |  |  | 0.89 (0.73-1.09) |
| HNC = head-neck cancer; OCC = oral cavity cancer; OHPC = oro- and hypopharyngeal cancer; LC = laryngeal cancer; Q = quartile; C = category  ^a^ Nutrient-density-adjusted intakes  ^b^ Adjusted for age (years) and sex  ^c^ Non-consumers  ^d^ Intake >0 g/day  ^e^ Possible violation of the proportional hazards assumption; no statistically significant interaction with time (*p* value ≥ 0.05)  ^f^ Possible violation of the proportional hazards assumption; statistically significant interaction with time (*p* value < 0.05) | | | | | | | | | | | | | | | |

**Supplemental Figures**


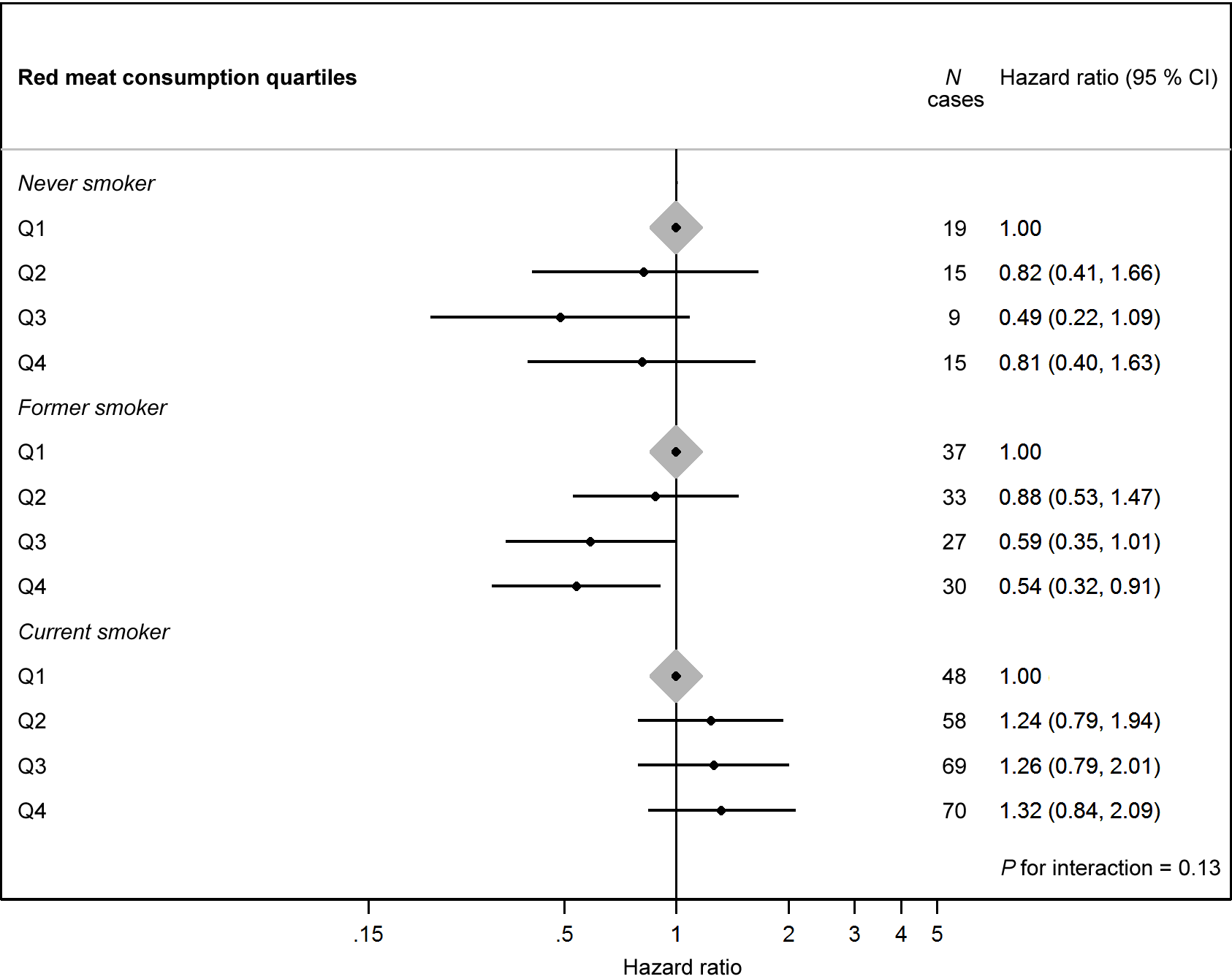


| **Supplemental Figure S1.** Red meat consumption stratified by smoking status: multivariate hazard ratios and 95 % confidence intervals for HNC (head-neck cancer) overall with adjustment for age (years), sex, frequency of smoking (numbers of cigarettes per day; centered), duration of smoking (number of years; centered), alcohol consumption (grams ethanol per day) and total energy intake (kcal/day) (Netherlands Cohort Study; 1986-2006) |
| --- |


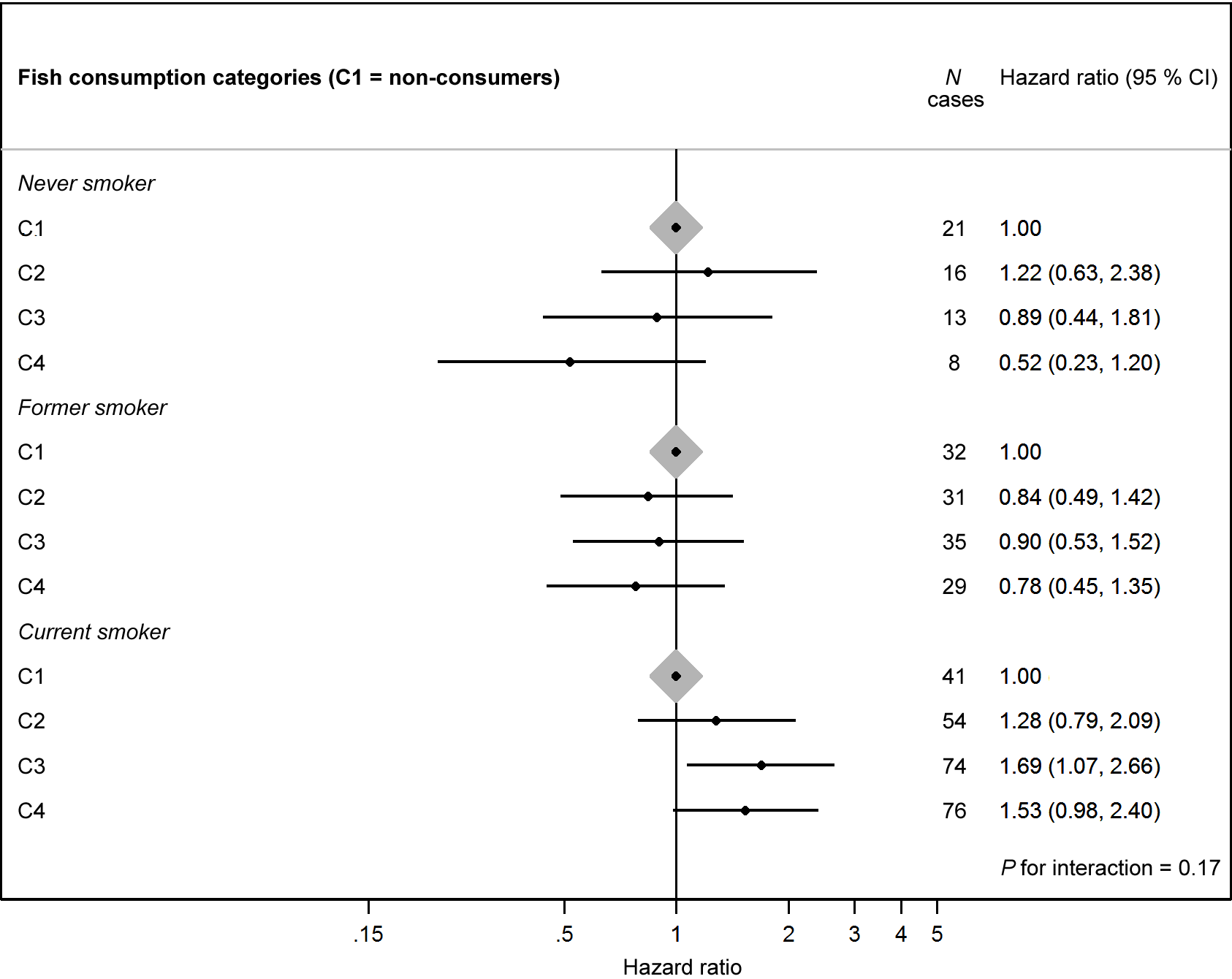


| **Supplemental Figure S2.** Fish consumption stratified by smoking status: multivariate hazard ratios and 95 % confidence intervals for HNC (head-neck cancer) overall with adjustment for age (years), sex, frequency of smoking (numbers of cigarettes per day; centered), duration of smoking (number of years; centered), alcohol consumption (grams ethanol per day) and total energy intake (kcal/day) (Netherlands Cohort Study; 1986-2006) |
| --- |


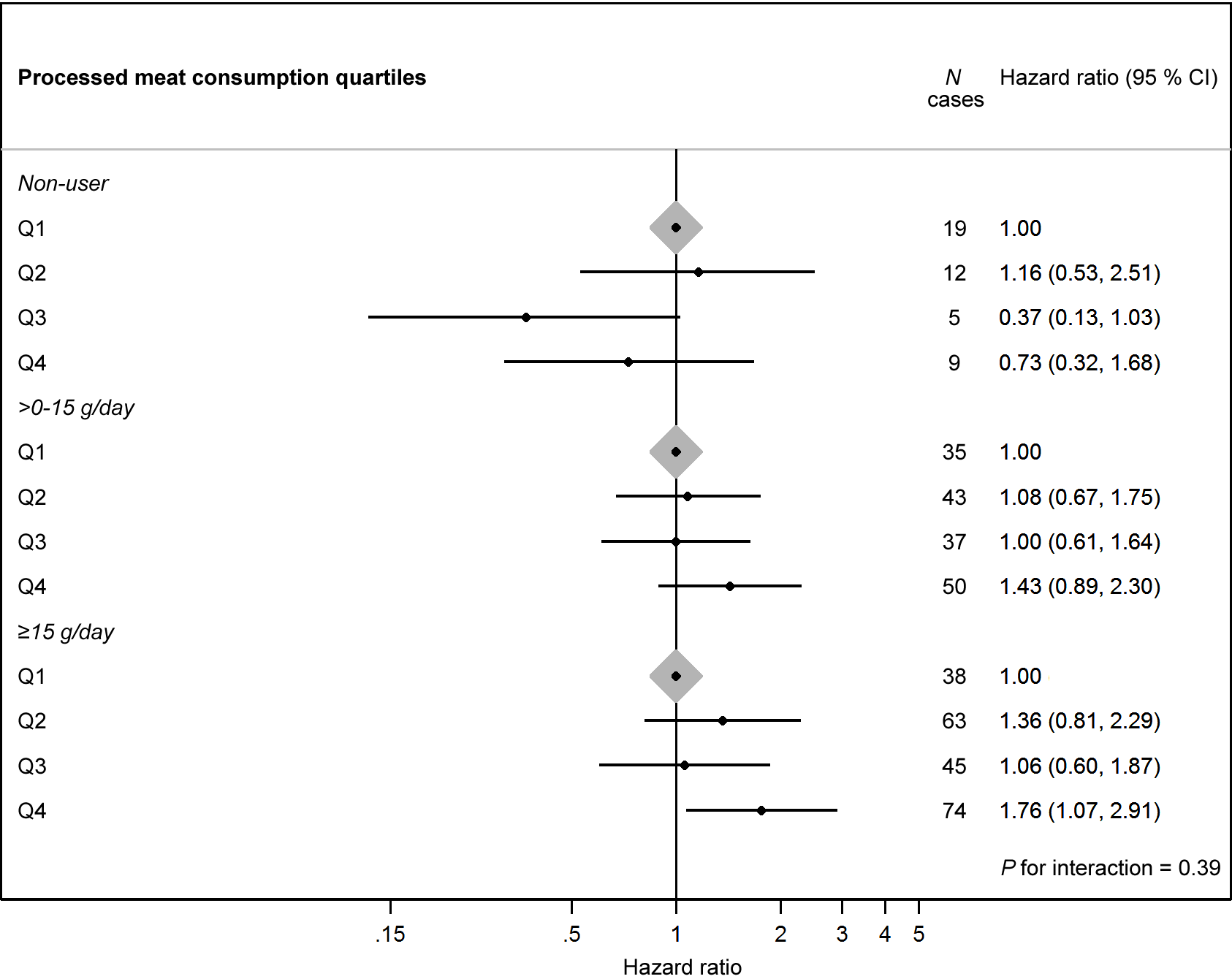


| **Supplemental Figure S3.** Processed meat consumption stratified by alcohol consumption: multivariate hazard ratios and 95 % confidence intervals for HNC (head-neck cancer) overall with adjustment for age (years), sex, cigarette smoking status (never/former/current), frequency of smoking (numbers of cigarettes per day; centered), duration of smoking (number of years; centered), alcohol consumption (grams ethanol per day) and total energy intake (kcal/day) (Netherlands Cohort Study; 1986-2006) |
| --- |


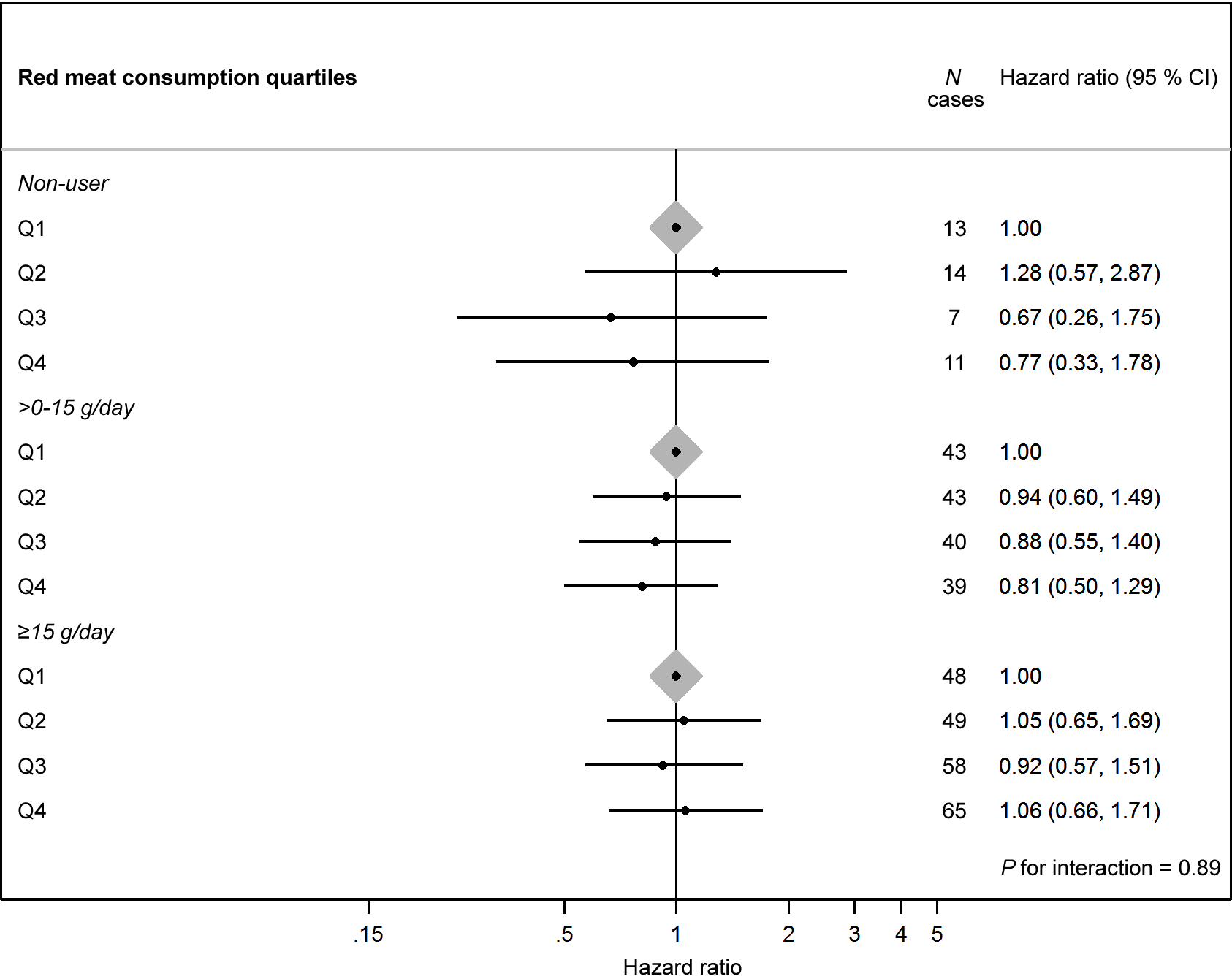


| **Supplemental Figure S4.** Red meat consumption stratified by alcohol consumption: multivariate hazard ratios and 95 % confidence intervals for HNC (head-neck cancer) overall with adjustment for age (years), sex, cigarette smoking status (never/former/current), frequency of smoking (numbers of cigarettes per day; centered), duration of smoking (number of years; centered), alcohol consumption (grams ethanol per day) and total energy intake (kcal/day) (Netherlands Cohort Study; 1986-2006) |
| --- |


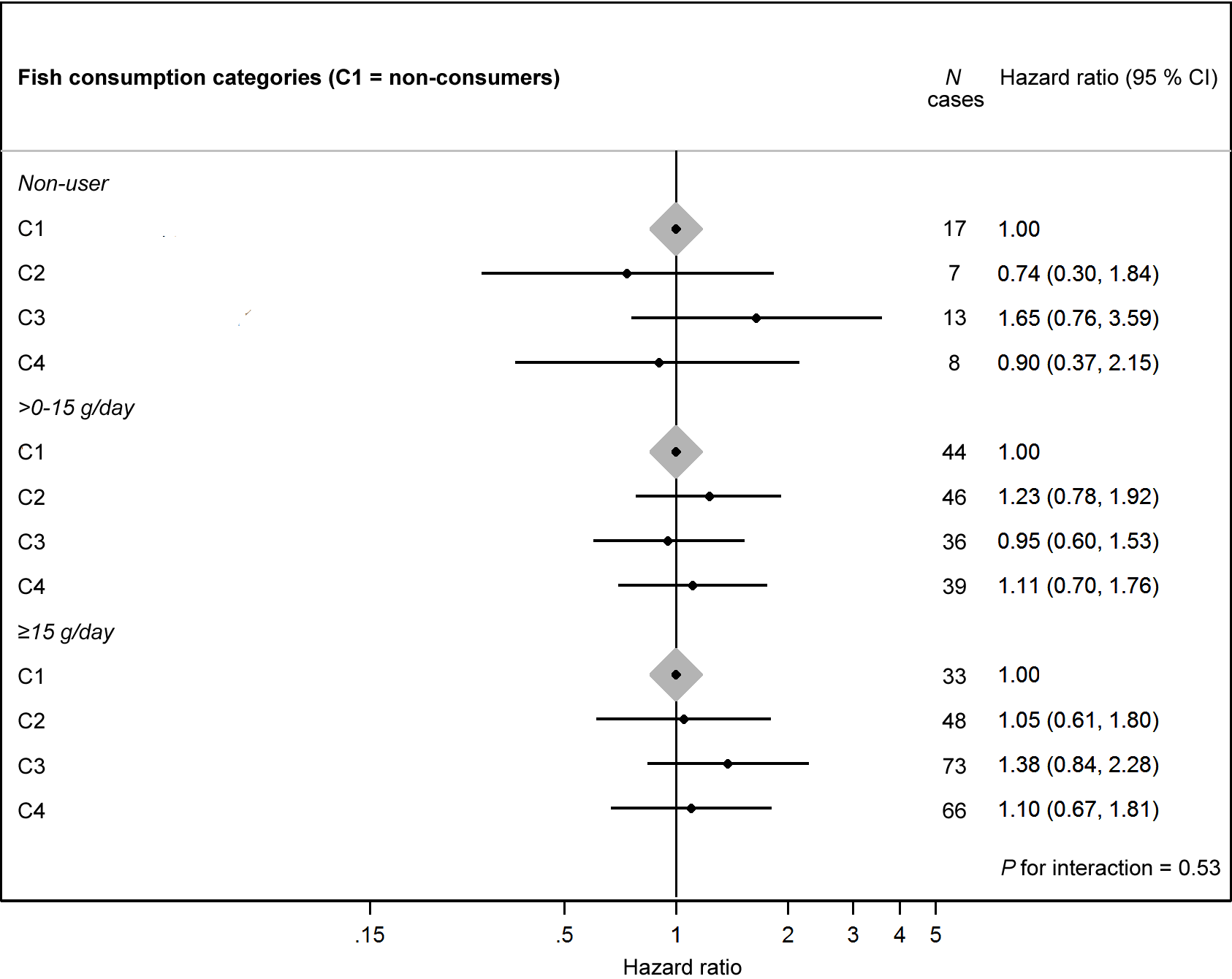


| **Supplemental Figure S5.** Fish consumption stratified by alcohol consumption: multivariate hazard ratios and 95 % confidence intervals for HNC (head-neck cancer) overall with adjustment for age (years), sex, cigarette smoking status (never/former/current), frequency of smoking (numbers of cigarettes per day; centered), duration of smoking (number of years; centered), alcohol consumption (grams ethanol per day) and total energy intake (kcal/day) (Netherlands Cohort Study; 1986-2006) |
| --- |


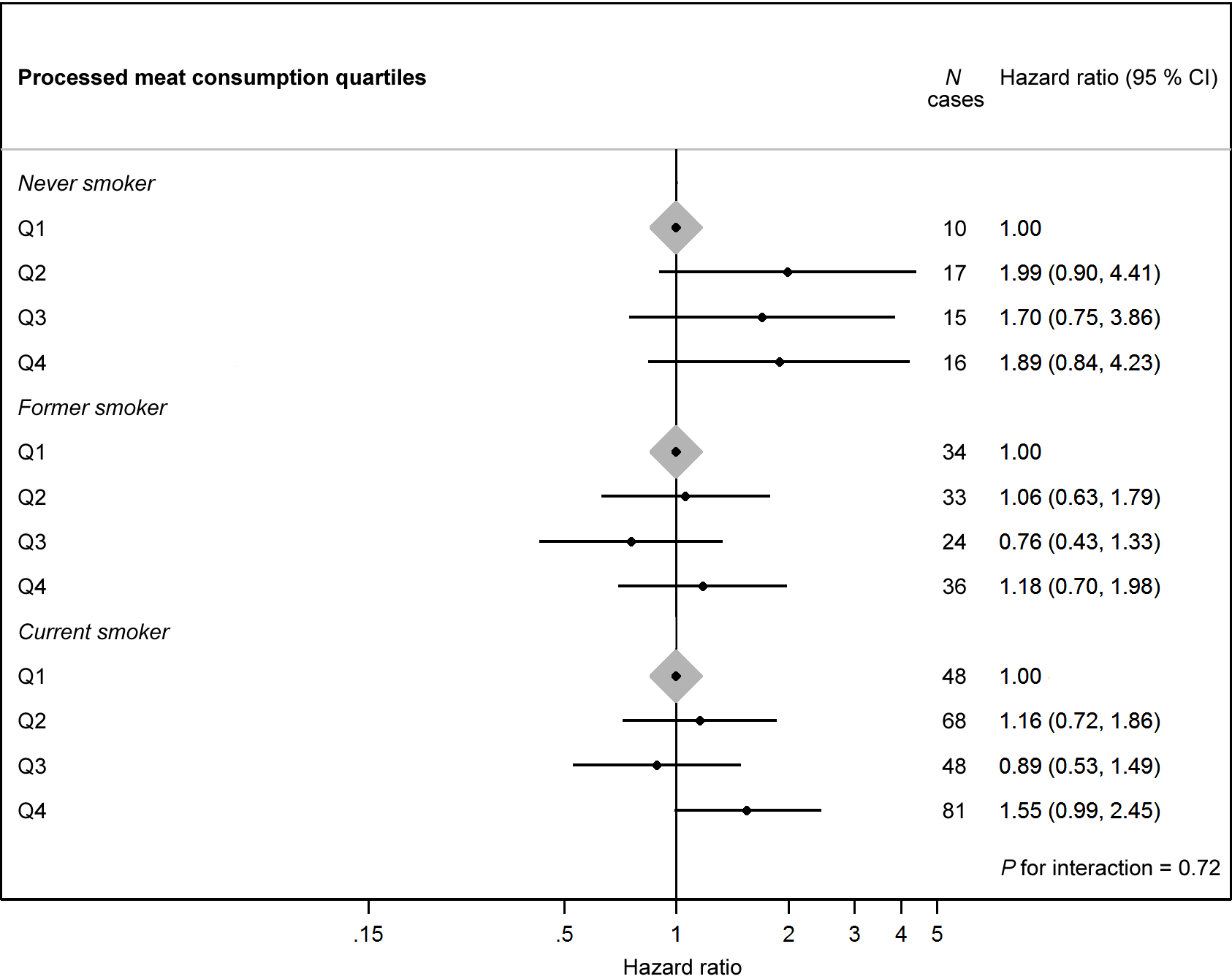


| **Supplemental Figure S6.** Processed meat consumption stratified by smoking status: multivariate hazard ratios and 95 % confidence intervals for HNC (head-neck cancer) overall with adjustment for age (years), sex, frequency of smoking (numbers of cigarettes per day; centered), duration of smoking (number of years; centered), alcohol consumption (grams ethanol per day) and total energy intake (kcal/day) (Netherlands Cohort Study; 1986-2006) |
| --- |
